# Wearable sensor systems measure differences in knee laxity between healthy and affected knees during dynamic exercise activities: A systematic review

**DOI:** 10.1002/jeo2.12094

**Published:** 2024-07-24

**Authors:** Sander C. van Eijck, Marly M. J. Vugts, Rob P. A. Janssen, Iris Hoogendoorn, Keita Ito, Maria C. van der Steen

**Affiliations:** ^1^ Department of Orthopedic Surgery & Trauma Máxima Medical Center Veldhoven The Netherlands; ^2^ Orthopedic Biomechanics, Department of Biomedical Engineering Eindhoven University of Technology Veldhoven The Netherlands; ^3^ Health, Innovations & Technology, Department of Paramedical Sciences Fontys University of Applied Sciences Eindhoven The Netherlands; ^4^ MMC Academy Máxima Medical Center Veldhoven Netherlands; ^5^ Department of Orthopedic Surgery & Trauma Catharina Hospital Eindhoven The Netherlands

**Keywords:** anterior cruciate ligament, eHealth, knee, knee arthroplasty, knee instability, osteoarthritis, wearables

## Abstract

**Purpose:**

Knee laxity can be experienced as knee instability which may lead to a limitation in the activity of patients. Current methods to determine knee instability are performed in a static setting, which does not always correlate with dynamic knee laxity during activities. Wearables might be able to measure knee laxity in a dynamic setting and could be of added value in the diagnosis and treatment of excessive knee laxity. Therefore, the aim of this systematic review is to provide an overview of the wearables that have been developed and their ability to measure knee laxity during dynamic activities.

**Methods:**

The PRISMA guidelines for systematic reviews were followed. A literature search was conducted in EMBASE, PubMed and Cochrane databases. Included studies assessed patients with knee instability using a non‐invasive wearable sensor system during dynamic activity, with comparison to a reference system or healthy knees. Data extraction was performed by two authors via a predefined format. The risk of bias was assessed by The Dutch checklist for diagnostic tests.

**Results:**

A total of 4734 articles were identified. Thirteen studies were included in the review. The studies showed a great variety of patients, sensor systems, reference tests, outcome measures and performed activities. Nine of the included studies were able to measure differences in patients with knee instability, all including a tri‐axial accelerometer. Differences were not measurable in all parameters and activities in these studies.

**Conclusions:**

Wearables, including at least a tri‐axial accelerometer, seem promising for measuring dynamic knee laxity in the anterior‐posterior and mediolateral direction. At this stage, it remains unclear if the measured outcomes completely reflect the knee instability that patients experience in daily life.

**Level of Evidence:**

Level III.

**Abbreviations**
ACL: anterior cruciate ligament
ADL: activities of daily living
AP: anterior‐posterior
IKDC: International Knee Documentation Committee
KAM: knee abduction moment
KOOS: Knee Injury and Osteoarthritis Outcome Score
OA: osteoarthritis
PCL: posterior cruciate ligament
PRISMA: Preferred Reporting Items for Systematic Reviews and Meta‐ Analysis
PROMS: patient reported outcome measurements
PROSPERO: prospective register of systematic reviews
TKA: total knee arthroplasty

## INTRODUCTION

Knee instability due to excessive knee laxity can be a result of different factors, such as traumatic events causing ligament ruptures, osteoarthritis (OA), primary total knee arthroplasty (TKA) or neuromuscular disorders. Knee laxity is described as a passive response of a joint to an externally applied force or torque. Knee instability only occurs when a knee ‘gives way’ during activities. Therefore, a knee with excessive laxity can be stable due to neuromuscular control [36]. Current treatment depends on the cause of the instability and may consist of physiotherapy, ligament reconstruction or revision arthroplasty. Instability is reported by patients as a feeling of the knee giving way or buckling [17, 33, 47, 52]. Patients with a similar underlying pathology and knee laxity can experience a different degree of knee instability. Some patients with excessive knee laxity do not experience instability even in demanding sports involving cutting or pivoting, while others experience symptoms of instability during activities of daily living (ADL) [13].

Considering the importance of instability during the planning of treatment, it is relevant to determine the amount of instability that a patient experiences [1]. Instability is currently determined with patient history and patient‐reported outcome measurements (PROMs) such as the Knee Injury and Osteoarthritis Outcome Score (KOOS) and International Knee Documentation Committee (IKDC) Subjective Knee Form [9, 20]. The lack of specificity of PROMs regarding instability makes it difficult to use them for clinical decision‐making [9, 46, 47]. Physical examination tests such as the Lachman‐, pivot shift‐, varus stress‐, valgus stress‐, muscle strength‐ and the anterior/posterior drawer tests are used in clinical practice to test the function of ligaments and muscles around the knee, indicating excessive knee laxity [39, 44]. Limitations of these tests are the inter‐examiner variability and subjective interpretation and grading of results [33, 39]. Several mechanical measurement systems have been developed to eliminate subjective grading of static tests, such as the KT 1000 arthrometer, inertial sensors, electromagnetic systems, optical motion analysis systems and accelerometers [3, 6, 19, 22, 23, 30, 32, 51, 60]. However, the results of these physical examination tests and objective measurement devices do not always correlate with functional instability experienced by patients during daily life [14, 21, 47, 49]. A possible explanation might be that the physical and mechanical tests are statically performed while instability mostly occurs during dynamic exercise. Multiple factors, such as muscular strength, neuromuscular control and capsuloligamentous laxity, are involved in the stability of the knee joint during dynamic activities [43]. Although dynamic instability is currently difficult to quantify, it is one of the most important factors in determining the optimal treatment for the individual patient [1].

Wearables can be a solution in quantifying dynamic instability as they might be able to provide clinicians with data gathered during daily life activities. As such, wearables might optimise diagnosis, treatment strategies, rehabilitation and follow‐up of knee patients [1]. Most wearables used in movement sciences consist of one or more of the following hardware to measure movements: (1) potentiometer, (2) gyroscope, (3) accelerometer and (4) magnetometers. When mounted on an object, potentiometers use an electric voltage that changes when the object is turned or displaced. As a result, the sensor is able to determine the object's position. Accelerometers are able to measure the acceleration of an object in a certain direction. By integration of the measurements, the distance and velocity of the object in relation to a reference point can be calculated. Gyroscopes can measure the angular displacement, velocity of angulation and angular rate of an object. A magnetometer is able to determine the magnetic north and is, therefore, important to determine the initial position and the position during movement of the other sensors in order to make accurate measurements using the accelerometer and gyroscope [57]. There is no overview of the techniques that have been studied to measure knee laxity during dynamic exercise activities using wearables and the relationship with experienced knee instability. Therefore, the aim of this systematic review is to provide an overview of the currently available wearables that have been developed to measure knee laxity during active dynamic activities, their ability to measure dynamic knee instability and the potential to be used in daily life.

## METHODS

### Protocol and registration

This systematic review was performed in accordance with the Preferred Reporting Items for Systematic Reviews and Meta‐Analysis (PRISMA) [42, 53]. The PRISMA checklist for systematic reviews can be found in Appendix A. The study protocol was registered in the International Prospective Register of Systematic Reviews (PROSPERO; CRD42021258581).

### Search strategy

On 14 April 2023, an experienced and independent information specialist (IH) performed a systematic literature search in Embase, PubMed and Cochrane databases. The search strategy applied search items and synonyms concerning ‘sensors’ and ‘knee instability’. An exemplary Embase search string can be found in Appendix B. All published literature up to 14 April 2023, was considered eligible. Duplicate articles were removed in Endnote following the method of Bramer et al. [7].

### Eligibility criteria

Inclusion and exclusion criteria are shown in Table 1. Two researchers (Marly M. J. Vugts and Sander C. van Eijck) independently screened the titles and abstracts identified by the search using the Rayyan QCRI app [41]. A full‐text version of all eligible abstracts was reviewed by two authors (Marly M. J. Vugts and Sander C. van Eijck) and cross‐checked for potential additional references. Disagreement between reviewers was resolved by discussion or by consulting a third independent researcher (Maria C. van der Steen).

**Table 1:** Inclusion and exclusion criteria.

Inclusion criteria	Exclusion criteria
Studies investigating patients with knee laxity originating from a knee disorder.	Laxity measured in another joint than the knee.
Measurement of laxity during dynamic exercise activities.	Patient is not able to perform dynamic exercise activities independently due to underlying diseases.
Laxity measurements performed with a non‐invasive wearable.	Laxity caused by neurological or muscular disease.
Comparison with stable knees (e.g., contralateral or healthy control group).	Animal, cadaver or robot studies.
Patient Population: All ages.	Conference papers, white papers, abstracts, reviews.
Full‐length publication in a peer‐reviewed journal.	
Languages: English, German or Dutch.	

### Data extraction

Two authors independently (Marly M. J. Vugts or Maria C. van der Steen, Sander C. van Eijck) extracted the data of interest to the review question and research objectives based on a predefined data collection form. Extracted data of all included articles was discussed to clarify data if necessary. Study characteristics (including year, author and study design), population characteristics (e.g., age, sex, type of knee injury and number of injured knees), wearable characteristics (e.g., type of sensor, outcome measure and measured degree of freedom) and information of the performed activity were extracted from the included studies. Furthermore, data concerning the reference group (e.g., age and sex) and, if applicable, reference system (type of test, type of questionnaire/questions and outcome measure) was extracted.

### Data synthesis

Data synthesis focused on the characteristics of the included study (type of injury, performed dynamic activity and reference test) and the characteristics of the sensor (hardware design, measured degrees of freedom, sampling rate, body attachment, wireless and used outcome measurements). The ability of a wearable to measure dynamic knee laxity was addressed by assessing results on differences detected between healthy and injured knees. Therefore, findings were classified as a significantly tested difference (++), data presenting a difference but not significantly tested (+), significantly tested no difference (‐‐) or data suggesting no difference but not significantly tested (‐). Furthermore, data was synthesised with respect to the relation between the outcome measured with the wearable and a reference test for instability (e.g., subjective reported instability addressed via questionnaires, instrumented Lachman tests and visual observations). Results between the wearable measurements and reference tests were scored in the following matter: statistically investigated high correlation/ICC (++), data presenting a correlation/coefficient but not statistically tested (+), statistically tested no correlation/high coefficient identified (‐‐) or data suggesting no correlation/coefficient but not statistically tested (‐). Due to the heterogeneity of the studied devices and data, a meta‐analysis or best‐evidence synthesis of the results was not possible.

### Risk of bias

The risk of bias in each included study was assessed independently by two authors (Marly M. J. Vugts or Sander C. van Eijck and Maria C. van der Steen). The Dutch checklist for diagnostic tests recommended by Cochrane was used [40]. Items focused on selection of participants, validity of the reference test, blinding and performance of the reference test and reporting of missing test results (Table 2). The item on the validity of the reference test was divided into two questions. The first question was about the validity of the reference groups in stable knees. The second question was about the validity of the external reference tests. Each domain was scored as ‘item adequately addressed’ (+), ‘item not adequately addressed’ (‐), or ‘unclear’ (?) regarding the risk of bias. Disagreement between assessors about the risk of bias was resolved through discussion.

**Table 2:** Risk of bias.

Study	Valid patient selection	Validated reference test for knee instability	Defined control group without knee instability	Independent assessment of the tests	Reference test performed independent of result index test	Dropouts were reported and the reason explained
Eymann et al. [15]	?	?	+	?	+	+
Favre et al. [16]	?	NA	+	?	+	+
Ishii et al. [24]	?	NA	+	?	+	+
Khan et al. [26]	?	?	+	?	+	‐
Kvist [28]	?	?	?	?	+	+
Kvist [29]	+	+	+	?	+	+
Misu et al. [34]	?	NA	?	?	+	+
Na and Buchanan [38]	+	?	+	?	+	+
Roberts et al. [45]	?	?	+	?	+	+
Soeno et al. [50]	?	?	+	?	+	+
Wada et al. [55]	?	NA	+	?	+	+
Yoshimura et al. [58]	?	NA	+	?	+	+
Yoshimura et al. [59]	?	NA	+	?	+	+

*Note*: +, item adequately addressed; ‐, item not adequately addressed;?, unclear; NA, not applicable.

## RESULTS

### Study selection and risk of bias

**Figure 1:**
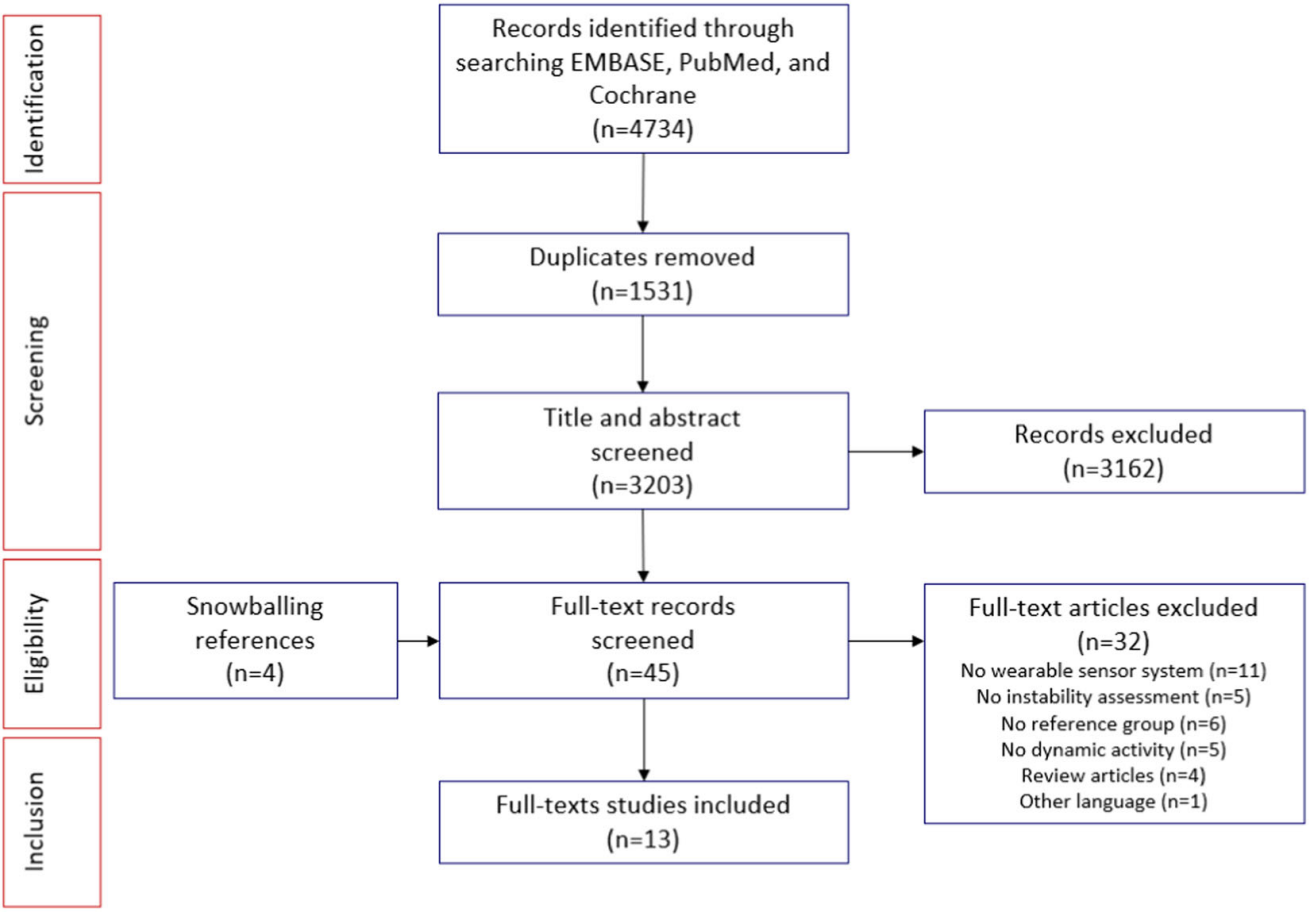
Preferred Reporting Items for Systematic Review and Meta‐Analyses (PRISMA) literature review flow diagram [35].

The Preferred Reporting Items for Systematic Review and Meta‐Analyses (PRISMA) flow diagram of the search is shown in Figure 1. A total of 13 articles were included in this systematic review after screening 4734 articles. 3162 articles were excluded during title/abstract screening as they did not meet inclusion/exclusion criteria. Another 32 articles were excluded for missing essential data (Figure 1).

Table 2 presents the risk of bias assessment of the included studies. Almost all articles (12/13) showed multiple potential sources of bias. The description of patient selection was limited in most studies. As there is no clear reference test for excessive knee laxity, most studies did not validate the used reference measurement. Almost all studies (12/13) reported dropouts from the study.

### Study characteristics

The characteristics of the various studies are presented in Table 3. In the 13 reviewed articles, a total of 420 patients were assessed for excessive knee laxity. The conditions studied for knee laxity were ligament injuries such as anterior and posterior cruciate ligament ruptures (ACL/PCL) [15, 16, 28, 29, 58, 59], laxity caused by OA [24, 34, 38, 55] or TKA [26, 45, 50]. The age of participants in studies focusing on ligament injuries was younger compared to studies focusing on TKA and OA.

**Table 3:** Study characteristics with respect to participants, activities and references*.*

Study	Patient characteristics								Dynamic activities performed												Reference						
	Condition			*N*	*N*	Age	*N*	*N*													Difference within subjects		Difference between subjects		Relation with other test method		
	Ligament injury	Total knee arthroplasty	Osteoarthritis	Participants	Knees	Years (Mean)	SD (years)	Male/female	Walking	Walking then sudden stop	Sit down then stand up	Step up and down a 7 inch box	Pivot (90° turn)	Active extension (with 0 then 8 kg)	Heel‐raising	Cycling (with 1.5 then 3.0 kg)	One legged squat	Chair squat (on two then one leg)	Drop jump	Single‐limb stance	Pre/postsurgery/intervention	Stable contralateral knee (*N*)	Healthy subjects (*N*)	Without subjective instability (*N*)	Questionnaire	Instrumented Lachman test	Visual assessment
Eymann et al. [15]	✓			33	66	43	‐	16/17									✓		✓			✓ (66)					✓
Favre et al. [16]	✓			5	‐	31.2	±6.7	‐	✓												✓	✓ (5)					
Ishii et al. [24]			✓	15	‐	64.9	±9.6	9/6	✓												✓		✓ (13)				
Khan et al. [26]		✓		27	38	50–80^a^	‐	11/16		✓	✓	✓	✓									✓ (12)	✓ (35)		✓		
Kvist [28]	✓			20	‐	29.3	±6.7	12/8	✓													✓			✓		
Kvist [29]	✓			12	‐	28	‐	8/4						✓	✓	✓	✓	✓				✓	✓ (17)			✓	
Misu et al. [34]			✓	16	32	62.8	±6.5	6/10	✓												✓		✓ (16)				
Na and Buchanan [38]			✓	26	‐	65.9	±6.1	10/16	✓														✓ (13)		✓		
Roberts et al. [45]		✓		27	38	66	±8	11/16		✓	✓	✓	✓										✓ (18)		✓		
Soeno et al. [50]		✓		76	16	74.2	‐	‐	✓															✓ (76)^b^	✓		
Wada et al. [55]			✓	68	68	69	‐	34/34	✓														✓ (68)				
Yoshimura et al. [58]	✓			40	80	24	‐	‐	✓													✓ (40)					
Yoshimura et al. [59]	✓			55	55	25–28^a^	‐	38/17	✓														✓ (60)				

*Note*: Characteristics of the patient (condition, mean age, distribution male/female, and body mass index), reference system, and activities performed during testing. ‐, not reported. ‘✓’ indicates activity which measured significant difference during dynamic activities. Abbreviation: *N*, *N*umber of participants; OA, osteoarthritis; TKA, total knee arthroplasty. Superscript ‘a’ indicates range instead of mean; ‘b’ indicates number of knees instead of participants.

^a^ Range instead of mean.

^b^ Number of knees instead of participants.

The performed dynamic exercise activities were diverse and aimed to mimic activities of daily living such as walking and cycling*.* Walking tests were performed most often (9/13) [16, 24, 28, 34, 38, 50, 55, 58, 59]. All of the included studies described the results of the wearable in comparison to either one or a combination of the following reference groups: (1) within subjects (comparing the injured knee with the contralateral healthy stable knee or comparing pre‐ and postsurgery results), (2) between subjects (a comparison with healthy subjects or subjects without subjective instability was made) and (3) a relation with other measurements (questionnaire, visual assessment or instrumented Lachman test).

### Wearable characteristics

Technical characteristics of the wearables are presented in Table 4. Four different hardware designs, often in combination, were used to measure knee laxity instability: (1) potentiometer, (2) gyroscope, (3) accelerometer and (4) magnetometers.

**Table 4:** Wearable characteristics.

	Gyroscope	Tri‐axial accelerometer	Tri‐axial gyroscope	Tri‐axial magnetometer	Uni‐axial accelerometer	Potentiometer	Number of sensors	Degrees of freedom	Sampling rate (Hertz)	Thigh and shank	Tibial tubercle	Lateral condyle femur	Foot	Tibia inferior to the tibial tubercle	Rubber/silicone strap	Strap/tape of other material	Tibial and femoral frame	Wireless	Instability in the transversal plane	Instability in the sagittal plane	Acceleration in m/s^2^	Degrees of motion in the knee joint	Translation in mm
Eymann et al. [15]		✓	✓	✓			1	9	200					✓	✓			✓	✓			✓	
Favre et al. [16]	✓						6	3	200	✓					✓			✓	✓			✓	
Ishii et al. [24]		✓	✓	✓			2		100		✓		✓		✓	✓		✓		✓	✓		
Khan et al. [26]		✓					1	3	100		✓				✓				✓		✓		
Kvist [28]						✓	4		2000	✓							✓		✓			✓	✓
Kvist [29]						✓	4		2000	✓							✓		✓			✓	✓
Misu et al. [34]		✓	✓				4		200		✓	✓			✓			✓		✓	✓		
Na and Buchanan [38]		✓	✓				2	6	NR					✓				✓	✓	✓	✓		
Roberts et al. [45]		✓	✓	✓			1	9	100		✓					✓		✓	✓	✓	✓		
Soeno et al. [50]		✓					1		250		✓				✓			✓	✓	✓	✓		
Wada et al. [55]		✓					4		1000			✓	✓	✓	✓	✓		✓	✓	✓	✓		
Yoshimura et al. [58]					✓		1		‐		✓					✓				✓	✓		
Yoshimura et al. [59]					✓		1		‐		✓					✓				✓	✓		

*Note*: ✓, Jerk was included as the outcome unit next to m/s^2^.

The number of sensors ranged between 1 and 6. All studies attached at least one sensor to the tibia. The direction of laxity was reported in the anterior/posterior direction (sagittal plane) and mediolateral direction for varus/valgus/medial/lateral trust or knee abduction moment (KAM) in the transversal plane. Reported outcome measures were mostly related to acceleration (9/13) [24, 26, 28, 29, 34, 38, 45, 54, 55, 58, 59] followed by degree of motion of the knee (4/13) [15, 16, 28, 29] and translation (2/13) [28, 29].

### Measurement of knee laxity

Results of the data synthesis classifying the results obtained with the wearables in relation to their ability to identify laxity differences and correlations with knee instability and reference tests are presented in Table 5. Nine studies showed a significant difference between the injured knee and the healthy knee on a variety of laxity‐related parameters [24, 26, 29, 34, 38, 45, 54, 55, 58, 59]. Three studies presented data suggesting that the wearable could measure excessive knee laxity but this was not statistically tested between groups [15, 16, 28]. One study found no measurable differences between patients with and without subjective knee instability, suggesting that it was not possible to measure knee laxity [50].

**Table 5:** Probability to measure dynamic knee laxity.

Study	Difference within subjects		Difference between subjects		Correlation with reference test		
	Pre/postsurgery/intervention	Healthy contralateral knee	Healthy subjects	Without subjective instability	Questionnaire	Instrumented Lachman test	Visual observation of instability
Eymann et al. [15]		+^a^					‐
Favre et al. [16]	+	+					
Ishii et al. [24]	++	++					
Khan et al. [26]		‐	++^a^		‐		
Kvist [28]		+			+		
Kvist [29]		++^a^	++^a^			‐	
Misu et al. [34]			++^a^				
Na and Buchanan [38]			++		++^a^		
Roberts et al. [45]			++^a^		+^a^		
Soeno et al. [50]				‐‐	‐		
Wada et al. [55]			++^a^				
Yoshimura et al. [58]	++^a^	++^a^	++^a^				
Yoshimura et al. [59]		++^a^					‐

*Note*: ++, Statistically significant measurable differences/correlation between groups in all directions; +, presented data suggests a difference/correlation between groups, but this is not statistically tested. ‐, presented data suggests no difference/correlation between groups, but this is not statistically tested; ‐‐, differences/correlations are statistically not significant for all measured directions.

^a^ Difference/correlation measurable but not on all parameters or activities.

In all studies that were able to measure excessive knee laxity, the wearable contained at least an accelerometer [24, 26, 28, 34, 38, 45, 54, 55, 58, 59]. Differences between normal knees and knees with excessive laxity were most often detected during walking [24, 34, 38, 55, 58, 59], followed by the activity of stepping up and down a box [26, 45], the pivot turn [26, 45] and drop jump [15].

In addition to a comparison with a reference group of stable knees, seven studies used an external reference test. Four studies looked into subjective stability compared to the results of the wearable [26, 38, 45, 50], of which three studies found a correlation between subjective instability and wearable outcomes [38, 45]. Only one study compared the Lachman test with the reference test and did not find a correlation [29]. Details about the included studies are described in Appendix C.

## DISCUSSION

The most important finding of this review is that the majority of the studies reported differences in knee laxity and stability between healthy and injured knees with wearable sensors. It seems possible to measure knee laxity during dynamic exercises using a wearable system in the anterior–posterior direction and mediolateral direction. The measured differences are often associated with differences in acceleration. There is limited evidence to what extend the measured differences reflect subjective instability experienced by the patients in daily life. This can be due to the fact that it is currently challenging to pinpoint the exact movements in which patients experience instability in daily life and the pitfalls of currently used methods in the anamnesis and physical examination to determine instability [56]. This might be the reason that all studies used at least a healthy control or healthy contralateral knee as a reference to determine if there is a difference in stability. Over time, the included studies show a decrease in sensor size and an improvement in the wireless usability of the sensor, which is important for possible future use in clinical practice. Although it seems possible to measure excessive dynamic knee laxity, current techniques are not well enough developed to be used in daily clinical practice.

A total of eight studies looked into differences in knee laxity between healthy controls, and a similar number of studies compared wearable outcomes with the healthy contralateral knee, while three studies looked at knee laxity pre‐ and posttreatment. The directions of laxity measured in all studies were in the transverse (anterior–posterior) and sagittal plane (mediolateral direction). Notably, rotatory laxity was not yet addressed in the currently available studies. Six studies detected differences in acceleration with a tri‐axial accelerometer which seems the most promising method for detecting knee instability in the AP and mediolateral direction [24, 26, 34, 38, 45, 55]. Translation in the AP direction in millimetres is an alternative, as was done by the studies of Kvist et al., which showed a significant difference between injured and healthy knees [28, 29]. It is worth mentioning that most studies assumed that a healthy knee does not have excessive laxity. This assumption could influence results as there are differences in knee laxity within the general population [5, 37]. However, due to the absence of a gold standard, a healthy (contralateral) knee seems a logical reference system. Nevertheless, extra validation seems preferable and is done in seven studies [15, 26, 28, 29, 38, 45, 50]. Five of these studies used questionnaires, and one study used a visual assessment. The Lachman test, which is often performed in the clinical setting, was used in one study to validate the measured knee laxity [8].

The study of Kvist et al. was the only study that looked into the correlation between the Lachman test and the measurements of the wearable, but they did not find a correlation [29]. A possible cause for the absence of a correlation between the physical examination tests and dynamic measurements could be that these tests are performed in a static setting. In a dynamic setting, muscles around the knee play an important role in achieving knee stability as well as proprioception and knee stiffness [56]. In the current review, none of the studies compared the dynamic measurements with collateral ligament tests or the pivot shift test, while this latter test for rotational knee laxity seems to correlate most with subjective instability complaints [4, 27]. Several studies showed that it is possible to measure acceleration in the transversal plane in a static, dynamic test setting during the pivot shift test, which correlated with the grading of the pivot shift test. This suggests that it might be possible to measure the pivoting movement also in a dynamic active setting [3, 6, 19, 23, 25]. Four of these studies used the same accelerometer to measure the Pivot shift [3, 6, 19, 25]. It would be interesting to see if the rotational component of this test is also measurable. Furthermore, it would be valuable to investigate if there is a relation between these measurements and subjective instability.

Subjective instability in relation to wearable measurements was studied in five of the included studies. The study of Na et al. showed a significant correlation, while the studies of Kvist and Roberts suggested a relation [26, 28, 38, 45]. Roberts et al. was the only study which presented the direction of instability (AP and mediolateral) in correlation with questionnaires [45]. Soeno and Khan did not find a correlation between subjective instability and measurement results [26, 50]. The finding that not all studies showed a significant correlation or no correlation with experienced instability could be due to several factors. First, the number of participants who experienced subjective instability taking part in the different studies was small, which could make a statistically significant difference difficult to measure [38, 45, 50]. Second, all sensors were used for a short duration in a controlled clinical or laboratory setting in which patients performed a limited number of activities. Patients might not have experienced instability during the measurements, but they might experience instability in daily life. Lastly, it is not exactly known which kind of instability causes subjective instability, and as a result, it is unknown if the sensors measured this direction of instability [56]. It is possible that subjective instability is more experienced in other directions such as pivoting movements that are known to cause instability but not yet studied [2].

For future research, it would be interesting to test wearable sensors in daily living over a longer period of time to quantify knee instability [47]. Results obtained in a laboratory setting can lack ecological validity. In other words, similar activities might still differ when tested in real life, as is seen in risk movement patterns within ACL patients [11, 12]. However, wearables showed promising results in a controlled clinical or laboratory setting. Factors such as size, weight, user‐friendliness, pairing with other devices and number of sensors will influence the usability of the wearable when applied in daily living. In this review, Kvist et al. used a frame, while all other studies used a rubber strap or tape to attach the wearable to the leg to improve usability. Elastic knee braces have also been used to attach wearables to measure knee range of motion [56, 57]. However, this might influence results for measuring knee instability because such a brace can act as an external stabilator [56, 57]. The influence of skin movements and muscle contractions could cause skin artefacts and influence the measurements of the sensors when attached to a strap [18, 45]. Future research should look into the frequencies of acceleration and rotation in relation to excessive knee laxity data in combination with the natural frequencies and skin artefacts of the IMU to confirm that the measurements are able to differentiate between measurement errors and knee laxity. None of the included studies looked into measurement variability due to skin artefacts. However, Roberts et al. performed a repeatability analysis, which showed no significant differences between these types of measurements regarding acceleration [45]. This is in accordance with the study by Liikavainio et al. who found no significant repeatability errors for peak acceleration and magnitude of acceleration when measuring gait with IMUs [31]. Other medical specialities used even further incorporated wearables, which might further increase reliability and usability by integrating the sensors in, for example, clothing, patches and tattoos [10]. Reported problems are mainly within data accuracy, implementation, regulation and affordability in current healthcare payment policies [10]. Furthermore, in future research, it would be interesting to compare wearable measurements with other movement analysis techniques, such as 3‐dimensional motion analysis. These systems are already used to measure knee instability and are already tested in healthy persons [6, 12, 48]. To make studies more replicable and comparable, a validated standardised physical examination, questionnaire and 3D motion analysis could assist as a more reliable reference test for future research.

This review has several limitations. Only studies in which the measurements of the wearable sensor system in an injured knee population were compared to a reference test or with stable knees were included. As such, studies that used a previously validated wearable sensor system but had no reported reference system or group were excluded. Consequently, sensor systems that were validated in a previous study and were found suitable to measure knee instability were not found in this review. Furthermore, only peer‐reviewed published studies were analysed. As a result of the positive publication bias, it might be that additional research has been performed in this field but was never published due to unfavourable results. Due to the lack of uniformity in sensor systems and study protocols, a coarse, although systematic interpretation of the results was provided in this review and meta‐analysis or best‐evidence evaluation was not possible.

## CONCLUSION

Wearables, including at least a tri‐axial accelerometer, seem promising for measuring dynamic knee laxity in the anterior‐posterior and mediolateral direction. At this stage, it remains unclear if the measured outcomes completely reflect the knee instability that patients experience in daily life.

## AUTHORS CONTRIBUTIONS

**Sander C. van Eijck**: Study design; study selection; data extraction; data interpretation; manuscript. **Marly M. J. Vugts**: Study design; study selection; data extraction; data interpretation; manuscript. **Rob P. A. Janssen**: Study design; study selection; data interpretation; manuscript. **Iris Hoogendoorn**: Search; study design; manuscript. **Keita Ito**: Study design; manuscript. **Maria C. van der Steen**: Study design; study selection; data extraction; data interpretation; manuscript.

## CONFLICT OF INTEREST STATEMENT

The authors declare no conflict of interest.

## ETHICS STATEMENT

Not applicable.

## Data Availability

Data that support the findings of this study are available from the corresponding author, Sander C. van Eijck, upon reasonable request.

## Appendix A

**Table:**

Section and Topic	Item #	Checklist item	Location where item is reported
**Title**			
Title	1	Identify the report as a systematic review.	Title page
**Abstract**			
Abstract	2	See the PRISMA 2020 for Abstracts checklist.	‐
**Introduction**			
Rationale	3	Describe the rationale for the review in the context of existing knowledge.	Page 3–4
Objectives	4	Provide an explicit statement of the objective(s) or question(s) the review addresses.	Page 3–4
**Methods**			
Eligibility criteria	5	Specify the inclusion and exclusion criteria for the review and how studies were grouped for the syntheses.	Page 5
Information sources	6	Specify all databases, registers, websites, organisations, reference lists and other sources searched or consulted to identify studies. Specify the date when each source was last searched or consulted.	Page 5
Search strategy	7	Present the full search strategies for all databases, registers and websites, including any filters and limits used.	Appendix
Selection process	8	Specify the methods used to decide whether a study met the inclusion criteria of the review, including how many reviewers screened each record and each report retrieved, whether they worked independently, and if applicable, details of automation tools used in the process.	Page 5
Data collection process	9	Specify the methods used to collect data from reports, including how many reviewers collected data from each report, whether they worked independently, any processes for obtaining or confirming data from study investigators, and if applicable, details of automation tools used in the process.	Page 5–6
Data items	10a	List and define all outcomes for which data were sought. Specify whether all results that were compatible with each outcome domain in each study were sought (e.g., for all measures, time points, analyses), and if not, the methods used to decide which results to collect.	Page 6
	10b	List and define all other variables for which data were sought (e.g. participant and intervention characteristics, funding sources). Describe any assumptions made about any missing or unclear information.	Page 6
Study risk of bias assessment	11	Specify the methods used to assess the risk of bias in the included studies, including details of the tool(s) used, how many reviewers assessed each study and whether they worked independently, and if applicable, details of automation tools used in the process.	Page 6
Effect measures	12	Specify for each outcome the effect measure(s) (e.g., risk ratio, mean difference) used in the synthesis or presentation of results.	Page 6
Synthesis methods	13a	Describe the processes used to decide which studies were eligible for each synthesis (e.g., tabulating the study intervention characteristics and comparing against the planned groups for each synthesis [item #5]).	Page 6
	13b	Describe any methods required to prepare the data for presentation or synthesis, such as handling missing summary statistics or data conversions.	Page 6
	13c	Describe any methods used to tabulate or visually display the results of individual studies and syntheses.	Page 6
	13d	Describe any methods used to synthesise results and provide a rationale for the choice(s). If meta‐analysis was performed, describe the model(s), method(s) to identify the presence and extent of statistical heterogeneity, and software package(s) used.	Page 6
	13e	Describe any methods used to explore possible causes of heterogeneity among study results (e.g., subgroup analysis, meta‐regression).	Page 6
	13f	Describe any sensitivity analyses conducted to assess the robustness of the synthesised results.	N/A
Reporting bias assessment	14	Describe any methods used to assess the risk of bias due to missing results in a synthesis (arising from reporting biases).	Page 6
Certainty assessment	15	Describe any methods used to assess certainty (or confidence) in the body of evidence for an outcome.	Page 6
**Results**			
Study selection	16a	Describe the results of the search and selection process, from the number of records identified in the search to the number of studies included in the review, ideally using a flow diagram.	Page 7
	16b	Cite studies that might appear to meet the inclusion criteria but which were excluded, and explain why they were excluded.	Page 7
Study characteristics	17	Cite each included study and present its characteristics.	Page 8
Risk of bias in studies	18	Present assessments of risk of bias for each included study.	Page 8
Results of individual studies	19	For all outcomes, present, for each study: (a) summary statistics for each group (where appropriate) and (b) an effect estimate and its precision (e.g., confidence/credible interval), ideally using structured tables or plots.	Page 9‐10
Results of syntheses	20a	For each synthesis, briefly summarise the characteristics and risk of bias among contributing studies.	Page 8‐10
	20b	Present results of all statistical syntheses conducted. If meta‐analysis was done, present for each the summary estimate and its precision (e.g., confidence/credible interval) and measures of statistical heterogeneity. If comparing groups, describe the direction of the effect.	N/A
	20c	Present results of all investigations of possible causes of heterogeneity among study results.	Page 8
	20d	Present results of all sensitivity analyses conducted to assess the robustness of the synthesised results.	N/A
Reporting biases	21	Present assessments of risk of bias due to missing results (arising from reporting biases) for each synthesis assessed.	Page 8 Table 2
Certainty of evidence	22	Present assessments of certainty (or confidence) in the body of evidence for each outcome assessed.	Page 11
**Discussion**			
Discussion	23a	Provide a general interpretation of the results in the context of other evidence.	Page 12‐14
	23b	Discuss any limitations of the evidence included in the review.	Page 12‐14
	23c	Discuss any limitations of the review processes used.	Page 14
	23d	Discuss the implications of the results for practice, policy, and future research.	Page 12‐14
**Other information**			
Registration and protocol	24a	Provide registration information for the review, including the register name and registration number, or state that the review was not registered.	Page 5
	24b	Indicate where the review protocol can be accessed, or state that a protocol was not prepared.	Page 5
	24c	Describe and explain any amendments to information provided at registration or in the protocol.	N/A
Support	25	Describe sources of financial or non‐financial support for the review and the role of the funders or sponsors in the review.	Title page
Competing interests	26	Declare any competing interests of review authors.	Title page
Availability of data, code and other materials	27	Report which of the following are publicly available and where they can be found: template data collection forms; data extracted from included studies; data used for all analyses; analytic code; any other materials used in the review.	Page 7–9 Appendix

## Appendix B

Search strings Embase.com database./exp = EMtree keywords with explosion./de = EMtree keywords without explosion.: ti,ab,kw = words in title, abstract of author keywords./it = publication type.

**Table:**

No.	Query
#5	#1 AND (#2 OR (#3 AND #4)) NOT ‘conference abstract’/it
#4	'knee’/exp OR ‘patellofermoral joint’ OR ‘articulatic genus’:ti,ab,kw OR ‘genopath*‘:ti,ab,kw OR ‘knee*‘:ti,ab,kw OR ‘knee compartment*‘:ti,ab,kw OR ‘knee joint*‘:ti,ab,kw OR ‘knee movement*‘:ti,ab,kw OR ‘knee stiffness*‘:ti,ab,kw OR ‘tibiofibular joint*‘:ti,ab,kw OR ‘patellofemoral joint*‘:ti,ab,kw
#3	'joint instability’/exp OR ‘joint laxity’/exp OR ‘joint stability’/exp OR ‘patellofemoral instability’/exp OR ‘arthrochalas*‘:ti,ab,kw OR ‘joint hyperextensibilit*‘:ti,ab,kw OR ‘joint hypermobilit*‘:ti,ab,kw OR ‘joint instabilit*‘:ti,ab,kw OR ‘joint laxit*‘:ti,ab,kw OR ‘joint stabilit*‘:ti,ab,kw OR ‘knee hyperextensibilit*‘:ti,ab,kw OR ‘knee hypermobilit*‘:ti,ab,kw OR ‘knee instabilit*‘:ti,ab,kw OR ‘knee laxit*‘:ti,ab,kw OR ‘knee stabilit*‘:ti,ab,kw OR ‘lax joint*‘:ti,ab,kw OR ‘lax knee*‘:ti,ab,kw
#2	(‘genu recurvatum’/exp OR ‘knee arthritis’/exp OR ‘knee disease’/de OR ‘knee injury’/exp OR ‘knee osteoarthritis’/exp OR ‘knee pain’/exp OR ‘knee surgery’/exp OR ‘valgus knee’/exp OR ‘varus knee’/exp OR ‘patellofemoral instability’/exp OR ‘acl injur*‘:ti,ab,kw OR ‘acl reconstruction*‘:ti,ab,kw OR ‘acl repair*‘:ti,ab,kw OR ‘acl rupture*‘:ti,ab,kw OR ‘acl surger*‘:ti,ab,kw OR ‘acl tear*‘:ti,ab,kw OR ‘anterior cruciate ligament injur*‘:ti,ab,kw OR ‘anterior cruciate ligament reconstruction*‘:ti,ab,kw OR ‘anterior cruciate ligament repair*‘:ti,ab,kw OR ‘anterior cruciate ligament rupture*‘:ti,ab,kw OR ‘anterior cruciate ligament surger*‘:ti,ab,kw OR ‘anterior cruciate ligament tear*‘:ti,ab,kw OR ‘bow leg*‘:ti,ab,kw OR ‘femorotibial arthros*‘:ti,ab,kw OR ‘genu extrorsum*‘:ti,ab,kw OR ‘genu introrsum*‘:ti,ab,kw OR ‘genu recurvatum*‘:ti,ab,kw OR ‘genu valgu*‘:ti,ab,kw OR ‘genu varum*‘:ti,ab,kw OR ‘genua valga*‘:ti,ab,kw OR ‘genua vara*‘:ti,ab,kw OR ‘gonarthrit*‘:ti,ab,kw OR ‘gonarthros*‘:ti,ab,kw OR ‘gonectypos*‘:ti,ab,kw OR ‘iliotibial band syndrom*‘:ti,ab,kw OR ‘knee arthrit*‘:ti,ab,kw OR ‘knee arthroplasty*‘:ti,ab,kw OR ‘knee arthros*‘:ti,ab,kw OR ‘knee disease*‘:ti,ab,kw OR ‘knee dislocation*‘:ti,ab,kw OR ‘knee disorder*‘:ti,ab,kw OR ‘knee injur*‘:ti,ab,kw OR ‘knee joint arthrit*‘:ti,ab,kw OR ‘knee joint arthros*‘:ti,ab,kw OR ‘knee joint dislocation*‘:ti,ab,kw OR ‘knee joint injur*‘:ti,ab,kw OR ‘knee joint osteoarthrit*‘:ti,ab,kw OR ‘knee joint replacement*‘:ti,ab,kw OR ‘knee joint trauma*‘:ti,ab,kw OR ‘knee ligament injur*‘:ti,ab,kw OR ‘knee ligament lesion*‘:ti,ab,kw OR ‘knee ligament reconstruction*‘:ti,ab,kw OR ‘knee ligament rupture*‘:ti,ab,kw OR ‘knee ligament surger*‘:ti,ab,kw OR ‘knee ligament trauma*‘:ti,ab,kw OR ‘knee meniscal fracture*‘:ti,ab,kw OR ‘knee meniscal injur*‘:ti,ab,kw OR ‘knee meniscal lesion*‘:ti,ab,kw OR ‘knee meniscal rupture*‘:ti,ab,kw OR ‘knee meniscus fracture*‘:ti,ab,kw OR ‘knee meniscus injur*‘:ti,ab,kw OR ‘knee meniscus lesion*‘:ti,ab,kw OR ‘knee meniscus rupture*‘:ti,ab,kw OR ‘knee open injur*‘:ti,ab,kw OR ‘knee osteoarthrit*‘:ti,ab,kw OR ‘knee osteo‐arthrit*‘:ti,ab,kw OR ‘knee osteoarthros*‘:ti,ab,kw OR ‘knee osteo‐arthros*‘:ti,ab,kw OR ‘knee pain*‘:ti,ab,kw OR ‘knee reconstruction*‘:ti,ab,kw OR ‘knee replacement*‘:ti,ab,kw OR ‘knee surger*‘:ti,ab,kw OR ‘knee trauma*‘:ti,ab,kw OR ‘knee varus*‘:ti,ab,kw OR ‘knock knee*‘:ti,ab,kw OR ‘meniscal allograft*‘:ti,ab,kw OR ‘meniscal repair*‘:ti,ab,kw OR ‘meniscal resection*‘:ti,ab,kw OR ‘meniscal surger*‘:ti,ab,kw OR ‘meniscal transplantation*‘:ti,ab,kw OR ‘meniscectomia*‘:ti,ab,kw OR ‘meniscus allograft*‘:ti,ab,kw OR ‘meniscus damage*‘:ti,ab,kw OR ‘meniscus removal*‘:ti,ab,kw OR ‘meniscus repair*‘:ti,ab,kw OR ‘meniscus resection*‘:ti,ab,kw OR ‘meniscus surger*‘:ti,ab,kw OR ‘meniscus tear*‘:ti,ab,kw OR ‘meniscus transplantation*‘:ti,ab,kw OR ‘menisectom*‘:ti,ab,kw OR ‘mescopath*‘:ti,ab,kw OR ‘osteoarthritis of the knee*‘:ti,ab,kw OR ‘painful knee syndrome*‘:ti,ab,kw OR ‘patella dislocation*‘:ti,ab,kw OR ‘patella luxation*‘:ti,ab,kw OR ‘patella sublucation*‘:ti,ab,kw OR ‘patellar dislocation*‘:ti,ab,kw OR ‘patellofemoral instabilit*‘:ti,ab,kw OR ‘pcl injur*‘:ti,ab,kw OR ‘pcl reconstruction*‘:ti,ab,kw OR ‘pcl repair*‘:ti,ab,kw OR ‘pcl rupture*‘:ti,ab,kw OR ‘pcl surger*‘:ti,ab,kw OR ‘pcl tear*‘:ti,ab,kw OR ‘posterior cruciate ligament injur*‘:ti,ab,kw OR ‘posterior cruciate ligament reconstruction*‘:ti,ab,kw OR ‘posterior cruciate ligament repair*‘:ti,ab,kw OR ‘posterior cruciate ligament rupture*‘:ti,ab,kw OR ‘posterior cruciate ligament surger*‘:ti,ab,kw OR ‘posterior cruciate ligament tear*‘:ti,ab,kw OR ‘torn menisc*‘:ti,ab,kw OR ‘valgus knee*‘:ti,ab,kw OR ‘varus knee*‘:ti,ab,kw) AND
#1	'accelerometer’/exp OR ‘accelerometry’/exp OR ‘goniometer’/exp OR ‘goniometry’/de OR ‘gyroscope’/exp OR ‘inertial sensor’/exp OR ‘magnetometer’/exp OR ‘magnetometry’/exp OR ‘motion analysis system’/exp OR ‘motion analysis’/exp OR ‘motion sensor’/exp OR ‘wearable device’/exp OR ‘wearable sensor’/exp OR ‘activity tracker’/exp OR ‘3d investigator*‘:ti,ab,kw OR ‘3dnx*‘:ti,ab,kw OR ‘3space fastrak*‘:ti,ab,kw OR ‘accelerometer*‘:ti,ab,kw OR ‘accelero‐meter*‘:ti,ab,kw OR ‘accelerometr*‘:ti,ab,kw OR ‘actical*‘:ti,ab,kw OR ‘activity ambulatory recorder*‘:ti,ab,kw OR ‘activity tracker*‘:ti,ab,kw OR ‘actiware ct*‘:ti,ab,kw OR ‘adxl322*‘:ti,ab,kw OR ‘articular arthrometr*‘:ti,ab,kw OR ‘articular goniometr*‘:ti,ab,kw OR ‘biomechanical function analysis system*‘:ti,ab,kw OR ‘dynaport minimod*‘:ti,ab,kw OR ‘egas*‘:ti,ab,kw OR ‘electromagnetic system*‘:ti,ab,kw OR ‘electromyographic sensor*‘:ti,ab,kw OR ‘electronic wearable device*‘:ti,ab,kw OR ‘electronic skin*‘:ti,ab,kw OR ‘genea*‘:ti,ab,kw OR ‘goniometer*‘:ti,ab,kw OR ‘goniometric measurement*‘:ti,ab,kw OR ‘goniometr*‘:ti,ab,kw OR ‘gt1m*‘:ti,ab,kw OR ‘gt3x*‘:ti,ab,kw OR ‘gyroscope*‘:ti,ab,kw OR ‘fitness tracker*‘:ti,ab,kw OR ‘imu*‘:ti,ab,kw OR ‘imu sensor*‘:ti,ab,kw OR ‘imus*‘:ti,ab,kw OR ‘inertia measurement unit*‘:ti,ab,kw OR ‘inertia measuring unit*‘:ti,ab,kw OR ‘inertia measuring device*‘:ti,ab,kw OR ‘inertia sensor*‘:ti,ab,kw OR ‘inertial measurement unit*‘:ti,ab,kw OR ‘inertial measuring device*‘:ti,ab,kw OR ‘inertial measuring unit*‘:ti,ab,kw OR ‘inertial sensor*‘:ti,ab,kw OR ‘intertial sensor*‘:ti,ab,kw OR ‘kinect*‘:ti,ab,kw OR ‘kinesiology ambulatory monitor*‘:ti,ab,kw OR ‘kinesiology ambulatory recorder*‘:ti,ab,kw OR ‘kinesiology sensor*‘:ti,ab,kw OR ‘kinetic activity monitor*‘:ti,ab,kw OR ‘kira*‘:ti,ab,kw OR ‘magnetometer*‘:ti,ab,kw OR ‘magnetometr*‘:ti,ab,kw OR ‘motion analysis system*‘:ti,ab,kw OR ‘motion capture device*‘:ti,ab,kw OR ‘motion capture system*‘:ti,ab,kw OR ‘motion detecting device*‘:ti,ab,kw OR ‘motion detector*‘:ti,ab,kw OR ‘motion node*‘:ti,ab,kw OR ‘motion sensing device*‘:ti,ab,kw OR ‘motion sensor*‘:ti,ab,kw OR ‘motion tracking*‘:ti,ab,kw OR ‘motionwatch*‘:ti,ab,kw OR ‘myomotion*‘:ti,ab,kw OR ‘optotrak*‘:ti,ab,kw OR ‘polaris*‘:ti,ab,kw OR ‘pro cmm*‘:ti,ab,kw OR ‘smart knee*‘:ti,ab,kw OR ‘smart‐e*‘:ti,ab,kw OR ‘vicon*‘:ti,ab,kw OR ‘wearable device*‘:ti,ab,kw OR ‘wearable electronic device*‘:ti,ab,kw OR ‘wearable sensor*‘:ti,ab,kw OR ‘wearable system*‘:ti,ab,kw OR ‘wearable technolog*‘:ti,ab,kw OR ‘wearables*‘:ti,ab,kw OR ‘xsens*‘:ti,ab,kw

## Appendix C

Details about the included studies are described in alphabetical order in the following paragraphs.

Eymann compared a visual assessment by an investigator to a wearable‐based movement assessment during multiple activities. As part of this study, sensor‐based inward tilt during a drop jump and squat test was compared between knees with a ligament injury and the contralateral knee as a measure of laxity of the knee joint. The authors describe a different inward angle during both tasks between the healthy and injured knee, with a difference between healthy knees and control knees during the drop jump; it is unclear if and how. There were only low intraclass correlations between the results of the wearable and visual reference tests [15]. Because the authors described a difference in laxity between the healthy knee, injured knee and contralateral knee but did not statistically test this, a +^a^ is reported in Table 5. The described correlation between the visual observation and test results was low, but it is unclear how this correlation was exactly determined, which results in a ‐ in Table 5 for the relation with the reference test.

Favre et al. used the range of motion (flexion/extension, internal/external rotation and abduction/adduction) during walking to differentiate between healthy knees and ACL insufficient and ACL reconstructed knees [16]. They found a significant difference in the range of motion between the ACL insufficient and ACL reconstructed knees as well as the healthy knees compared to the ACL insufficient knees. The authors did not report specific measurements regarding the instability of the knee. Considering the finding that the difference in ROM between the healthy and injured knee as well as pre‐and postoperative ROM is described but not in relation with instability a + is reported in Table 5.

Ishii et al. investigated lateral trust in patients with knee OA during walking [24]. The authors added after the first trial a wedge insole in the shoe to investigate if this would decrease lateral trust. They found a higher peak lateral acceleration between the control group and the knee OA group during walking without an insole. With a wedge insole in the shoe, there was no difference in acceleration (lateral trust) between the OA group compared to the control group. Because the study showed a significant difference in acceleration between healthy knees and OA knees during walking without insole and between with and without insole ++ was scored twice in Table 5.

Khan et al. compared anterior–posterior acceleration in TKA knees with healthy knees during four different exercises (Table 3) [26]. A higher mean in the total magnitude of acceleration in the anterior–posterior direction was found in TKA patients compared to the control knees during stepping down and turning. There was no difference detectable during sit‐to‐stand, sudden stop and stepping up. There was no significant difference between the TKA knees and their contralateral knees nor between cruciate retaining TKA and posterior stabilising TKA. Patients' responses on experienced instability during the activities did not correspond with the acceleration values. Considering the finding that there was a significant difference between TKA knees and control knees in two activities, a ++^a^ is reported in Table 5. There was no significant difference in all activities between the TKA and contralateral knee; therefore, a ‐ is presented for this parameter in Table 5. There was no relationship between the wearable results and instability complaints, but this was not statistically tested, which resulted in a ‐ in Table 5.

Kvist et al. assessed a poor‐functioning, non‐operated ACL‐injured group with a well‐functioning, nonoperated ACL‐injured group during walking while measuring dynamic translation of the tibia [28]. The Lysholm score was used to differentiate between the well‐functioning and poor‐functioning groups, with a cut‐off score of 84. There was a 24% greater anterior translation between the well‐functioning ACL group compared to the healthy contralateral knee. The poor‐functioning group had 16% less translation in the injured leg compared to the healthy contralateral knee. There was a significant difference in anterior–posterior translation between the poor‐functioning injured knees and well‐functioning injured knees. Because the difference between the healthy and injured knees was not explicitly statistically tested, we noted a + in Table 5. For the relationship with the Lysholm score, we looked at the finding that the group with a low Lysholm score had a different translation compared to the group with a high Lysholm score. However, this was not statistically tested in relation to the questionnaire, and, therefore, a + is scored in Table 5.

In another study, Kvist et al. compared ACL insufficient knees with their contralateral knee and healthy controls during different exercises [29]. There was a significantly larger maximum translation in ACL insufficient knees compared to the contralateral knee, as well as healthy controls during several but not all exercises. There was no correlation reported between the translation during the static Lachman test and the translation during the dynamic tests. All types of activities—but not for all conditions—were able to detect a difference between contralateral knees and healthy control knees, which resulted in two ++ ^a^, as shown in Table 5. Because there was no statistical test reported regarding the absence of a correlation between the dynamic measurements and the Lachman test results this was scored a ‐.

Misu et al. used the root mean square (RMS) acceleration data of the tibia and femur in the mediolateral direction during the first half of the stance phase to assess joint trust. In addition, an adjusted RMS was calculated to correct for the swing speed during gait [34]. Both measures were significantly different between OA patients and healthy knees in the mediolateral direction when measured on the tibia. However, when adjusted for age and BMI, no significant association between knee OA and adjusted RMS was found. Since the difference in measurement was seen in all activities but was not visible after statistical correction: Table 5 ++^a^.

Na et al. compared OA knees with healthy knees during a 10‐m walk. They found a significant difference in acceleration and jerk (the rate of change of the knees' acceleration over time) in patients with OA compared to healthy control knees during a 10‐m walk [38]. The differences were the largest during the midstance of the gait cycle. This data was used to investigate the relation between self‐reported and dynamic instability. There was a significant relationship between linear acceleration and self‐reported instability but not for jerk and self‐reported instability. Patients experiencing more instability showed higher levels of acceleration. The instability was measured in the frontal, sagittal and transverse plane. The directions in which a significant difference was measurable are not described in the manuscript. Due to the fact that the study found a significant difference in acceleration and jerk between groups, we noted a ++ in Table 5. Because the linear acceleration correlated significantly with the questionnaires, jerk did not, and the relation was scored as ++^a^.

Roberts et al. investigated the mean, maximum, minimal and range of linear acceleration and jerk of the linear acceleration in the medial/lateral, anteroposterior and inferior/superior directions during five dynamic tasks (Table 3) [45]. In 22 of the 150 parameters, a higher value was found for TKA patients compared to healthy controls. Quantity, distributional measure and direction on which the significant difference was found differed between tasks. None of the significantly different parameters were related to the medial/lateral direction. According to the authors, the range and absolute acceleration in the anterior/posterior direction during the step‐up/step‐down activity proved to be the best indicators of knee laxity. This was also the activity in which subjective instability corresponded best with the wearable measurements. Subjective instability and/or pain did not correspond with the measurement outcomes during the other tasks. Considering the statistical difference between groups but not all tested parameters, a ++^a^ is presented in Table 5. As the link between the experienced instability and the outcome of the wearables was not statistically tested, this relation is presented as +^a^ in Table 5.

Soeno et al. performed a walking test for which TKA patients were divided into two groups (subjective stability and subjective instability) based on the results of a self‐made instability questionnaire [50]. They did not find a difference in RMS of acceleration and the cumulative amplitude of acceleration with an accelerometer during walking between the two groups in the vertical, anteroposterior and mediolateral directions. The authors concluded that there is no measurable difference between subjective stable and unstable knees with TKA. Because there was no difference in all activities and parameters between the groups, we reported a ‘‐‐’ in Table 5. Combining this fact together with a division between groups using questionnaires, no statistical test was performed between self‐reported knee instability score and outcome of the wearables; this is scored as a ‐ in Table 5.

Wada et al. used a stepping exercise to mimic stair climbing to induce muscle fatigue, followed by wearable measurements during walking [55]. The total acceleration, as well as the change in acceleration after stepping, were significantly larger in the OA group in the proximal‐distal, anterior–posterior and mediolateral direction compared to the control group. Furthermore, they found a significant correlation between OA patients with varus trust and anterior–posterior acceleration and an increased joint convergent angle. The tests were performed before and after exercises to see if muscle fatigue influenced the instability of the knee. The difference in accelerations increased when patients experienced muscle fatigue, considering the statistical difference between groups but not on all tested parameters: Table 5 ++^a^.

Yoshimura et al. investigated acceleration in ACL insufficient patients compared to ACL reconstructed knees and healthy knees during walking with a focus on lateral trust [58]. The peak value of acceleration was significantly larger in the ACL insufficient knees compared to ACL reconstructed knees and healthy knees when a lateral thrust pattern was identified. There was no significant difference in lateral acceleration between ACL reconstructed knees compared to healthy knees. ACL insufficient knees showed a significantly larger acceleration, suggesting more lateral trust when the injury was more than 3 years old compared to ACL insufficient knees with an injury less than 3 years ago. There was no difference detected in medial trust for all parameters [58]. Considering the fact that for one direction, it was possible to measure acceleration differences, a ++^a^ is presented in Table 5 for comparison with both groups.

Yoshimura et al. performed a similar study to measure acceleration with ACL and PCL insufficient knees during 10 gait cycles compared with healthy knees to assess lateral and medial trust [59]. The peak value of lateral acceleration was larger for ACL‐insufficient knees compared to healthy knees when a lateral thrust pattern was detected. The lateral acceleration was increased in the PCL insufficient group with lateral thrust patterns but this difference was not significantly different compared to healthy knees [59]. Medial trust was not significantly different between groups. Because lateral trust was significantly different, but medial trust, not a ++^a^ is reported in Table 5.
